# Systemic Immune-Inflammation Index: A Novel Predictor for Non-dipper Hypertension

**DOI:** 10.7759/cureus.28176

**Published:** 2022-08-19

**Authors:** Abdurrahman Akyüz, Ferhat Işık

**Affiliations:** 1 Department of Cardiology, University of Health Sciences Gazi Yaşargil Training and Research Hospital, Diyarbakır, TUR

**Keywords:** systemic immune-inflammation index, non-dipper, hypertension, dipper, arterial blood pressure

## Abstract

Introduction

The non-dipper hypertension (HT) pattern is associated with more end-organ damage and cardiovascular events than is dipper HT. Inflammation is widely established to play a role in the pathophysiology of HT. Recently, a new inflammatory and prognostic marker called the systemic immune-inflammation index (SII) has emerged. Our goal is to determine whether there is a relationship between non-dipper HT and SII.

Methods

Our study is a single-center retrospective and ninety-one patients with HT were included. All patients were analyzed with simultaneous 24-hour ambulatory blood pressure monitoring and laboratory parameters. Thirty-five patients had dipper HT while 56 patients had non-dipper HT. SII was calculated according to neutrophil, platelet, and lymphocyte counts.

Results

The median age was 48 (45-61 interquartile range (IQR)) in the non-dipper HT group, whereas it was 54 (44-64 IQR) in the dipper HT group. Although the neutrophil level, neutrophil-lymphocyte ratio, platelet lymphocyte ratio, SII, sleeping systolic blood pressure (BP), and sleeping diastolic BP were higher (p=0.020, p=0.041, p=0.046, p=0.019, p<0.001, and p=0.001, respectively) in the non-dipper HT group, the lymphocyte level was lower (p=0.040). A multivariate logistic regression model shows that SII (odds ratio (OR)=1.023, 95% confidence interval (CI)=1.002-1.112, p=0.012) may be an independent predictor of non-dipper HT.

Conclusion

Our study showed that the SII level was higher in the non-dipper HT patient group than in the dipper HT group. Furthermore, SII was an independent predictor of non-dipper HT. The high SII value in hypertension patients can be used as an early warning parameter to identify non-dipper HT patients.

## Introduction

Hypertension (HT) is a common chronic disease all over the world and is the most common risk factor for cardiovascular disease (CVD) [1]. Despite the use of various modalities for the treatment of HT, target organ damage has not been prevented to the desired level [2]. In hypertensive patients, systolic and diastolic blood pressure (BP) is expected to decrease by more than 10% during sleep compared to during the daytime, and these values are expected to show a circadian variation called dipper HT. In non-dipper HT, this circadian variation does not occur, and BP reductions are less than 10% [3]. Non-dipper HT has been linked to an increased risk of cardiac disease and target organ damage when compared to dipper HT [4,5]. In patients with non-dipper HT, the risk of atherosclerotic events is three times higher than that with dipper HT [6]. Inflammation is well-known to play a role in the pathophysiology of HT [7]. Inflammatory markers are associated with BP variability [8]. In both cancer and CVD, the systemic immune inflammation index (SII) computed from neutrophil, platelet, and lymphocyte counts is a significant prognostic predictor [9-12]. There are insufficient data to determine whether a new inflammatory parameter, SII, is associated with non-dipper HT. The aim of this study was to determine whether SII is an independent predictor in patients with non-dipper HT.

## Materials and methods

Patient selection

Our study was single-center and retrospective and included 91 consecutive hypertensive patients with 24-hour ambulatory blood pressure monitoring (ABPM). The patients were divided into two groups on their HT profiles. Thirty-five patients had dipper HT while56 patients had non-dipper HT. HT was defined as systolic BP ≥140 mmHg and/or diastolic BP ≥ 90 mmHg or previously diagnosed HT under antihypertensive medication. Chronic kidney disease, chronic liver disease, heart failure, history of coronary artery disease, anemia, acute or chronic infectious disease, inflammatory disease, malignancy, and secondary HT were excluded from the study. The study was carried out in conformity with the Helsinki Declaration [13], and the local ethics committee approved it. Ethics committee approval was obtained from the scientific research ethics committee of the University of Health Sciences, Diyarbakir Gazi Yaşargil Education and Research Hospital, dated April 21, 2022, approval number: 74.

ABPM records

All participants in this study were subjected to 24-hour ABPM. The patient's non-dominant arm was chosen for cuff installation. The 24-hour ABPM recordings were obtained every 15 minutes throughout the day and every 30 minutes at night. Awake and sleep times were calculated using the information provided by the patients. (percent) 100 x [1 - (sleep systolic BP/awake systolic BP)] was used to calculate nighttime BP reduction. Dipper HT was defined as a decline in systolic and diastolic blood pressures of greater than 10%. Non-dipper HT was characterized as a drop in systolic and diastolic blood pressure of less than 10% [3].

Laboratory analysis

Blood samples from all participants on the day of ABPM insertion were analyzed. The complete blood count was evaluated using a Beckman Coulter LH 750 analyzer (Galway, Ireland). The platelet/lymphocyte ratio (PLR) was calculated by dividing the platelet count by the lymphocyte count. The neutrophil/lymphocyte ratio (NLR) was obtained by dividing the neutrophil count by the lymphocyte count. SII was calculated using the formula SII = (neutrophil count x platelet count)/lymphocyte count [9].

Statistical analysis

The IBM SPSS software suite was used to conduct all statistical analyses (IBM SPSS Statistics for Windows, Version 24.0. Armonk, NY: IBM Corp.). Continuous variables were presented as mean±SD and median interquartile range (IQR) 25-75% in case of non-normal distribution. Categorical variables were expressed as percentages. Depending on the data distribution, continuous variables were compared using the student's t-test or the Mann-Whitney U test. The categorical variables were compared using chi-square or Fisher's exact tests when suitable. The Mann-Whitney U test was used to evaluate the non-normal distributed numerical and categorical variables in the two groups. Multivariable logistic regression analysis was performed to identify independent predictors of non-dipper HT. Logistic regression modeling was created with the parameters in Table 1 with p<0.2 (age, gender, serum creatinine, angiotensin-converting enzyme (ACE) inhibitors, angiotensin receptor blockers (ARBs), β-blockers, hemoglobin, and SII). A p-value of <0.050 was considered statistically significant. Receiver operating characteristic (ROC) curve analysis was performed to obtain the area under curve (AUC) of the SII for predicting non-dipper HT.

**Table 1:** Baseline demographic and clinical characteristics of dipper and non-dipper HT patients Abbreviations: HT: hypertension; ACE: angiotensin-converting enzyme, ARBs: angiotensin receptor blockers, BP: blood pressure, CCBs: calcium channels blockers, NLR: neutrophil to lymphocyte ratio, PLR: platelet to lymphocyte ratio, SII: systemic immune-inflammation index, WBC: white blood cell

	Dipper(n = 35)	Non-dipper(n = 56)	p-value
Age (years)	54(44-64)	48(45-61)	0.248
Gender (females), n (%)	20(57.1)	33(58.9)	0.867
Diabetes mellitus, n (%)	4(11.4)	11(19.6)	0.307
ACE inhibitors, n (%)	8(22.9)	23(41.1)	0.076
ARBs, n (%)	19(54.3)	18(32.1)	0.037
β-Blockers, n (%)	18(51.4)	16(28.6)	0.029
CCBs, n (%)	18(51.4)	23(41.1)	0.337
Diuretics, n (%)	21(60.0)	39(69.6)	0.348
Serum creatinine, mg/dL	0.77(0.68-0.99)	0.73(0.66-0.83)	0.091
WBC, ×103/mm3	7.96(6.28-9.15)	8.77(7.39-9.96)	0.123
Neutrophil, ×103/µL	4.35(3.69-6.31)	5.31(4.32-7.62)	0.020
Lymphocyte, ×103/µL	2.47(1.92-2.80)	2.14(1.59-2.62)	0.040
Platelet, ×103/µL	273(227-311)	283(245-334)	0.255
Hemoglobin, g/dL	14.1(13.2-15.4)	13.4(12.4-14.8)	0.109
NLR	1.76(1.51-2.66)	2.26(1.64-3.54)	0.041
PLR	106.6(88.0-138.9)	131.3(96.5-174.5)	0.046
SII	522.5(343.1-794.4)	662.7(475.8-1093.2)	0.019
Systolic BP (24 hours average) (mmHg)	127.0(119.0-135.0)	126.5(118.3-139.8)	0.500
Diastolic BP (24 hours average) (mmHg)	79.0(76.0-85.0)	78.0(72.3-85.8)	0.546
Systolic BP (awake) (mmHg)	131.0(125.0-139.0)	127.0(119.0-138.5)	0.175
Diastolic BP (awake) (mmHg)	83.0(80.0-87.0)	78.0(72.3-85.8)	0.093
Systolic BP (sleep) (mmHg)	109.0(103.0-119.0)	123.5(116.3-134.2)	<0.001
Diastolic BP (sleep) (mmHg)	66.0(56.0-78.0)	75.5(70.0-83.0)	0.001

## Results

Table 1 shows the comparison of basic demographic and clinical characteristics of dipper and non-dipper hypertensive patients. The median age was 48 (45-61 interquartile range (IQR)) in the non-dipper HT group, whereas it was 54 (44-64 IQR) in the dipper HT group. The use of angiotensin receptor inhibitors (ARBs) and beta-blockers was higher in the dipper HT group (p=0.037 and p=0.029). Although the neutrophil level, NLR, PLR, SII, sleeping systolic BP, and sleeping diastolic BP were higher (p=0.020, p=0.041, p=0.046, p=0.019, p<0.001, and p=0.001, respectively) in the non-dipper HT group, the lymphocyte level was lower (p=0.040).

A multivariable logistic regression model was performed using the parameters age, gender, serum creatinine, angiotensin-converting enzyme inhibitors, ARBs, beta-blockers, hemoglobin, and SII, to identify independent predictors of non-dipper HT (Table 2).

**Table 2:** Multivariable logistic regression analysis to determine predictors of non-dipper HT Abbreviations: HT: hypertension; ACE: angiotensin-converting enzyme, ARBs: angiotensin receptor blockers, SII: systemic immune-inflammation index

	OR	CI	p
Age (years)	1.005	0.957-1.055	0.850
Gender (females), n (%)	0.510	0.149-1.747	0.283
Serum creatinine, mg/dL	0.066	0.004-1.029	0.052
ACE inhibitors, n (%)	1.176	0.298-4.640	0.817
ARBs, n (%)	0.643	0.174-2.371	0.507
β-blockers, n (%)	0.638	0.224-1.817	0.400
Hemoglobin, g/dL	0.732	0.402-1.034	0.063
SII	1.023	1.002-1.112	0.012

**Figure 1:**
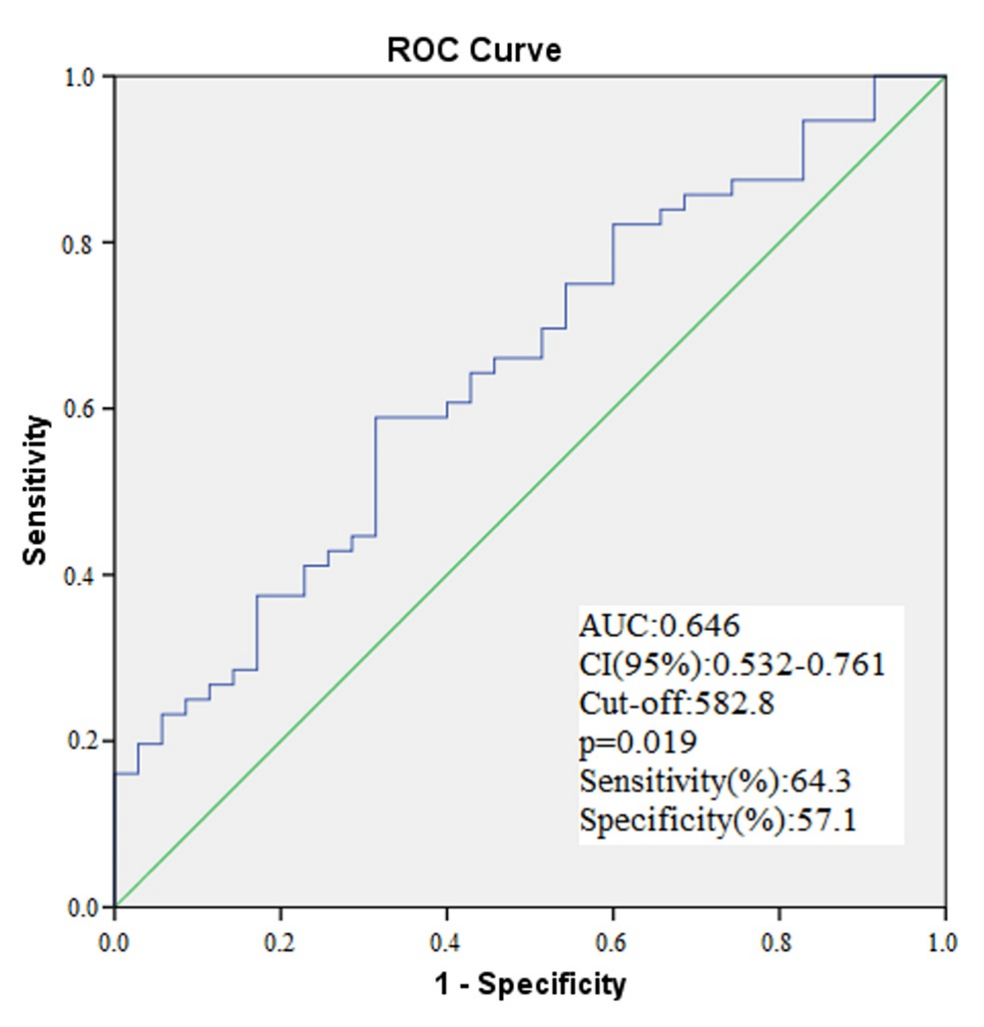
ROC analysis of SII performed to predict non-dipper HT Abbreviation: HT: hypertension; AUC: area under curve, CI: confidence interval

This model shows that SII (odds ratio (OR)=1.023, 95% confidence interval (CI)=1.002-1.112 p=0.012) may be an independent predictor of non-dipper HT. In the ROC analysis of SII performed to predict non-dipper HT, the optimal predicting value for SII was 582.8, with a 64.3% sensitivity and a 57.1% specificity. The AUC of SII was 0.646 (95% CI = 0.532-0.761) (Table 3, Figure 1).

**Table 3:** Receiver operating characteristic (ROC) curve comparison of SII level in predicting non-dipper HT Abbreviations: HT: hypertension; AUC: area under the ROC curve, SII: systemic immune-inflammation index

Risk factor	AUC(95%)	Cut off	p	Sensitivity(%)	Specificity(%)
SII	0.646(0.532-0.761)	582.8	0.019	64.3	57.1

## Discussion

Our study showed that the SII level was higher in the non-dipper HT group than in the dipper HT group. In addition, multivariate logistic regression analysis determined that SII was an independent predictor for non-dipper HT.

Individuals with non-dipper HT had a greater widespread inflammatory response, more severe end-organ damage, and increased cardiovascular morbidity and mortality, according to previous research [14-15]. The non-dipper BP pattern had a deleterious effect on cardiovascular risk regardless of whether the BP level was normal or above the usual range [16]. Non-dipper HT's deleterious effect may be due to endothelial injury. In a previous study, non-dipper HT patients had lower endothelial progenitor cell counts than dipper HT patients, which is important for endothelial homeostasis and vascular repair [17]. Chronic inflammation is associated with many chronic diseases such as chronic kidney disease, HT, diabetes mellitus, coronary artery disease, connective tissue disease, and malignancy [18-21]. Inflammation is linked to BP variability and plays an important role in the pathogenesis of HT. High BP variability, in particular, may cause vascular inflammation [22]. Kim et al. showed that inflammatory mediators, such as IL-6, high-sensitivity C-reactive protein, and TNF-α, are associated with BP variability [8]. Kawada et al. showed an independent relationship between neutrophils and HT [23]. In an animal study, Barhoumi et al. showed that regulatory T lymphocytes suppressed BP elevation and angiotensin-II-mediated vascular damage [24]. In hypertensive patients, there has been an observed increase in the aggregation tendency of platelets, which is induced by adenosine diphosphate [25]. This activation is caused by impairment of both L-arginine uptake and platelet nitric oxide production in hypertensive patients [26]. According to Kaya et al., platelet activation was increased in non-dipper hypertensive patients compared to dipper ones [27]. In our study, we found that neutrophil, NLR, PLR, and SII levels were higher while lymphocyte levels were lower in the non-dipper HT group, similar to the results of previous studies [14]. SII has emerged as a means of predicting the prognosis and consequences of cancer and heart patients using peripheral blood cells such as platelets, neutrophils, and lymphocytes [9,10]. In patients with coronary artery disease, SII has been demonstrated to be a reliable predictor of major adverse cardiovascular events [10]. Çırakçıoğlu et al. showed a significant relationship between SII and carotid intima-media thickness in hypertensive patients [28]. Furthermore, Saylik et al. found that SII levels were higher in recently diagnosed treatment-naive hypertension patients with exaggerated morning BP rises [29]. Inflammatory indicators have been extensively studied for their efficacy in predicting bad outcomes in high-risk HT patients. It is known that the risk of major cardiovascular events is higher in the hypertensive patient group, especially in those with a non-dipper HT pattern. In our study, we found that SII was higher in the non-dipper HT group and that a high SII level was an independent indicator of non-dipper HT. SII can be used as an easily calculated auxiliary marker for detecting non-dipper HT patients among those with high BP. SII may help in the creation of an early treatment approach to minimize complications in non-dipper HT patients who are at a higher risk of cardiovascular events.

The most important limitation of our study is that it is retrospective and the number of patients is small. Another limitation is that we could not evaluate the prognostic value of SII in patients with non-dipper HT.

## Conclusions

Our study showed that the SII level was higher in the non-dipper HT patient group than in the dipper one. Furthermore, SII was an independent predictor of non-dipper HT. The high SII value in hypertension patients can be used as an early warning parameter to identify non-dipper HT patients.
